# Using Small-scale Blast Tests and Numerical Modelling to Trace the Origin of Fines Generated in Blasting

**DOI:** 10.1007/s00501-018-0778-9

**Published:** 2018-09-25

**Authors:** Ivan Kukolj, Armin Iravani, Finn Ouchterlony

**Affiliations:** Chair of Mining Engineering and Mineral Economics, Montanuniversitaet Leoben, Erzherzog-Johann-Str. 3, 8700 Leoben, Austria

**Keywords:** Blast-induced fines, Blast tests, High-speed photography, FEM, DEM, Dynamic cracking, Blast fragmentation, Sprenginduzierte Feinanteile, Sprengversuche, Hochgeschwindigkeitsfotografie, FEM, DEM, Dynamische Rissbildung, Sprengzerkleinerung

## Abstract

Waste fines from rock breakage often negatively influence economics and environment. The Austrian Science Fund (FWF) sponsors a project to investigate the cause of the fines by studying blast fragmentation throughout small-scale blast tests and numerical simulations. The tests include blast-loading confined granite and mortar cylinders by detonating cord with 6, 12, and 20 g/m of PETN. The blast-driven dynamic cracking at the end face of the cylinder opposite to the initiation point is filmed with a high-speed camera. The filming is followed up by an analysis of surface and internal crack systems and sieving of the blasted cylinders to quantify the amount of fine material created. The numerical simulations cover the blast fragmentation of a mortar cylinder. These simulations use Finite and Discrete Element Methods (FEM, DEM) with explicit time integration. The model cylinders are loaded by a pressure evolution acting on the borehole wall. Both methods produce realistic crack patterns, consisting of through-going radial cracks with crack intersections around a crushed zone at the borehole. Furthermore, the DEM models have also yielded realistic fragment size distributions (FSD). The paper covers the present progress of the ongoing project and related future work.

## Zusammenfassung

Bei Gesteinssprengungen entstehende, nicht verwertbare Feinanteile haben oft einen negativen Einfluss auf Umwelt und Wirtschaftlichkeit. Der Österreichische Wissenschaftsfonds (FWF) fördert ein Projekt zur Untersuchung der Ursache von Feinanteilen durch Analyse der Zerkleinerung in kleinmaßstäblichen Sprengversuchen und numerische Modellierung. Die Tests umfassen Sprengversuche an Granit- und Mörtelzylindern mit 6, 12 und 20 g/m PETN Sprengschnur. Die dynamische Rissbildung an der dem Initiationspunkt gegenüberliegenden Stirnseite des Zylinders wird mit einer Hochgeschwindigkeitskamera aufgenommen. Im Anschluss an die Aufnahme erfolgt eine Analyse der oberflächlichen und inneren Risssysteme sowie eine Siebanalyse des gesprengten Materials, um die Masse des entstehenden Feinmaterials zu quantifizieren. Die numerischen Simulationen modellieren die Zerkleinerung eines Mörtelzylinders. Es werden Finite- und Diskrete-Elemente-Methoden (FEM, DEM) mit expliziter Zeitintegration verwendet. Die Modellzylinder werden durch eine, auf die Bohrlochwand wirkende, Druckentwicklung belastet. Beide Methoden produzieren realistische Rissmuster, bestehend aus durchgehenden radialen Rissen mit Rissübergängen (Vereinigung und Unterteilung) rund um eine Zermalmungszone am Bohrloch. Darüber hinaus haben die DEM-Modelle auch eine realistische Korngrößenverteilung geliefert. Der Beitrag behandelt den aktuellen Fortschritt des laufenden Projekts sowie damit verbundene zukünftige Arbeiten.

## Introduction

The amounts of mineral fines that are associated with raw materials extraction have practical consequences. One concern is the sustainability of natural resources since fines are often an unsellable liability or waste that has cost money and energy to produce and in the end has to be deposited. This reasoning lay behind the EU project Less Fines [1]. The health aspects of mineral fines have again come into focus, so sources of respirable dust and mitigation effects are studied in the ongoing EU Horizon 2020 project “Sustainable Low Impact Mining, SLIM” [2, 3].

Blasting is a major producer of waste fines, crushing and milling another. Blasting is a highly dynamic process and the crack growth that defines breakage is a major source of fines. Such crack-generated fines (CGF) are also produced by crushing and grinding. Fines are inherently related to the amount of energy required in comminution. Most of the area created resides in the fines and this area then determines the consumed comminution energy [4]. A better knowledge of how CGF are generated may also help to improve blasting and crushing practices and to suppress the amount of CGF at the source rather than dealing with them afterwards.

**Fig. 1:**
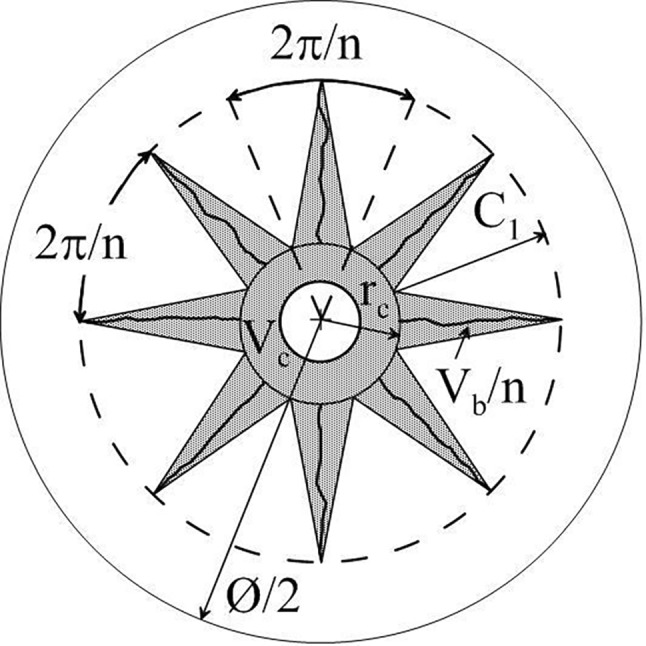
Volume of crushed material around an extended 2D blast-hole (Fig. 2 in [7]); the crushed zone volume V_c_ is annular and the breakage zone volume V_b_, which also generates crushed fines, is made up of the *n* partial volumes of the star arms

Blast generated fines are often considered to originate mainly from the annular crushed zone around a blast hole, which contains only −1 mm material, and fragmentation models were built around this; e. g. the CZM or crush zone model [5, 6]. It implies that fragment size is (almost) solely defined by the distance to the blast hole, the finest material created at the borehole wall and fragment size, thus, increasing with distance from the blast hole. The circular crushed-zone model was extended to a star-shape one (Fig. 1; [7]).

**Fig. 2:**
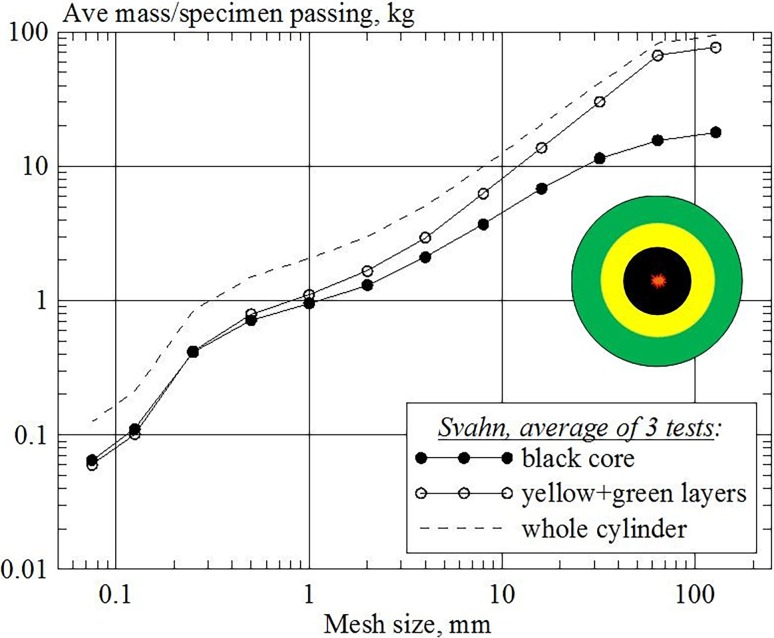
Sieving curves for blasted multilayered Ø300×600-mm cylinders of mortar; comparison of Ø120-mm *black core* with *yellow* (Ø120–200 mm) + *green layers* (Ø200–300 mm) [8]

Blasting tests with layered cylinder specimens [8] contradict the predictions of the star-shaped CZM (Fig. 2). Firstly, the sieving curves for the layers are quite similar in shape and the core region contains fragments well beyond 1 mm in size. Secondly, there is a cross-over point (0.25 mm in Fig. 2) above which the outer layers contain more fine material than the core. Consequently, more −1 mm fines are created outside the black core than inside it.

**Fig. 3:**
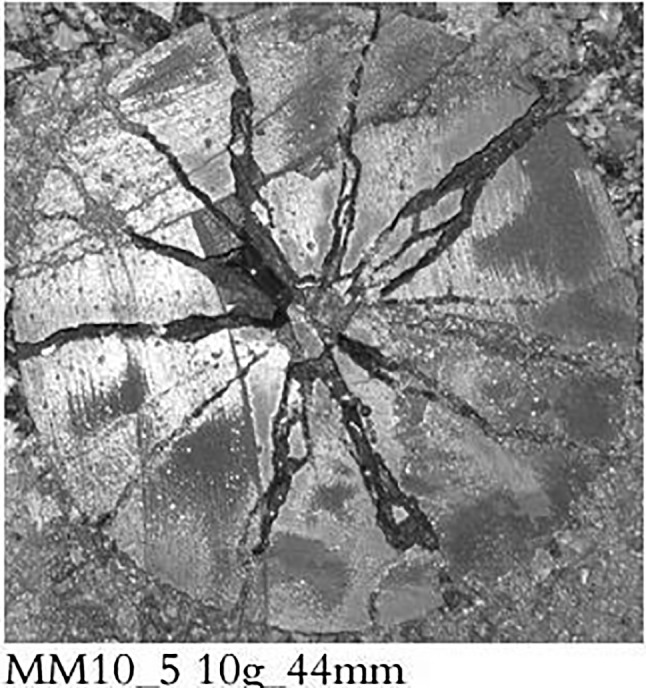
Post-blast cross section through blasted confined mortar cylinder [9]

Post-mortem crack patterns are not as simple either as the CZM (Fig. 3; [9]). Here the cracks have seemingly run along crooked paths, branched, merged, and left debris along the crack paths.

Statistical models of brittle fragmentation [10, 11] point in the same direction, that instability of fast propagating cracks leaves behind a trace of small fragments along their propagation paths, but this has not been observed in rock under blasting-like conditions. More arguments are provided in [12].

This led to the FWF-sponsored project P27594-N29: “Fines generated by dynamic crack propagation, as in blasting of rock-like materials,” which ends Dec 31, 2018. Two main project objectives are to: i) determine the importance of the dynamic mechanism for CGF by capturing images of branching at a moving crack tip and ii) compare the measured fragment size distribution (FSD) with models based either on the mechanism of crack branching and merging or other mechanisms. The first part of this project is described in [13, 14], progress in numerical simulations in [15], and the present state in this paper. It is divided into an experimental part and a part with numerical simulations.

## Methodology

### Small-scale Blast Tests

The blast tests [13] include controlled blast loading of a confined hollow cylinder whilst the resulting dynamic cracking is filmed at its frontal end face by means of high-speed photography.

The cylinder is made of mortar or granite, Ø150 × 300 mm in size, with a Ø10-mm central axial borehole. The production of the blast cylinders is described in [13].

The loading is achieved by detonating a decoupled PETN (*Pentaerythritol tetranitrate*) cord (6, 12, or 20 g/m) inside the borehole. The detonation propagates along the cord towards a stemming plug at the frontal end face with the velocity (VOD) of about 7300 m/s [16].

**Fig. 4:**
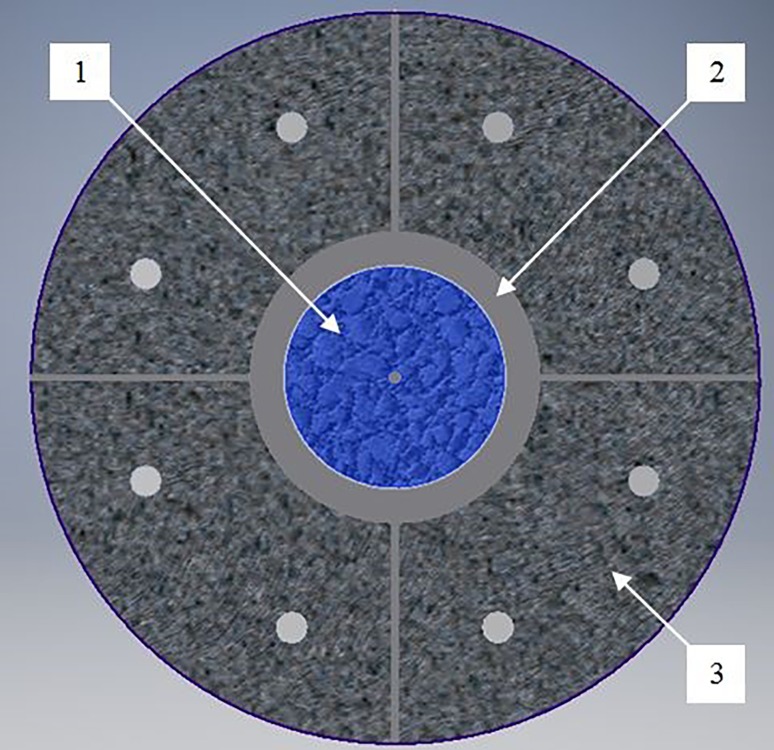
Prepared blast chamber (transverse cross-section) (*1*—Blast cylinder; *2*—Damping layer; *3*—Blast chamber)

The cylinder is radially confined by a 25-mm-thick damping layer inside a blast chamber (Fig. 4). The damping material [13] is a commercial concrete mixture, cured for one day. It improves the acoustic-impedance matching of propagating shock waves and protects the chamber.

Table 1 shows measured material properties of the blast cylinders and the damping layer.

**TABLE 1:** Measured material properties

Property	Granite		Mortar		Damping	
	Mean	St.dev	Mean	St.dev	Mean	St.dev
UCS [MPa]	171.50	9.00	27.70	1.10	–	–
Brazilian tensile strength [MPa]	10.85	1.52	2.90	0.49	–	–
Density [g/cm^3^]	2.70	0.01	1.66	0.01	2.12	0.08
Young’s modulus [GPa]	65.30	0.83	12.20	0.31	–	–
Poisson’s ratio [–]	0.24	0.02	0.23	0.05	–	–
*P*-wave velocity [m/s]	4908	111	3069	62	1210	274
*S*-wave velocity [m/s]	3212	150	2065	40	643	79

**Fig. 5:**
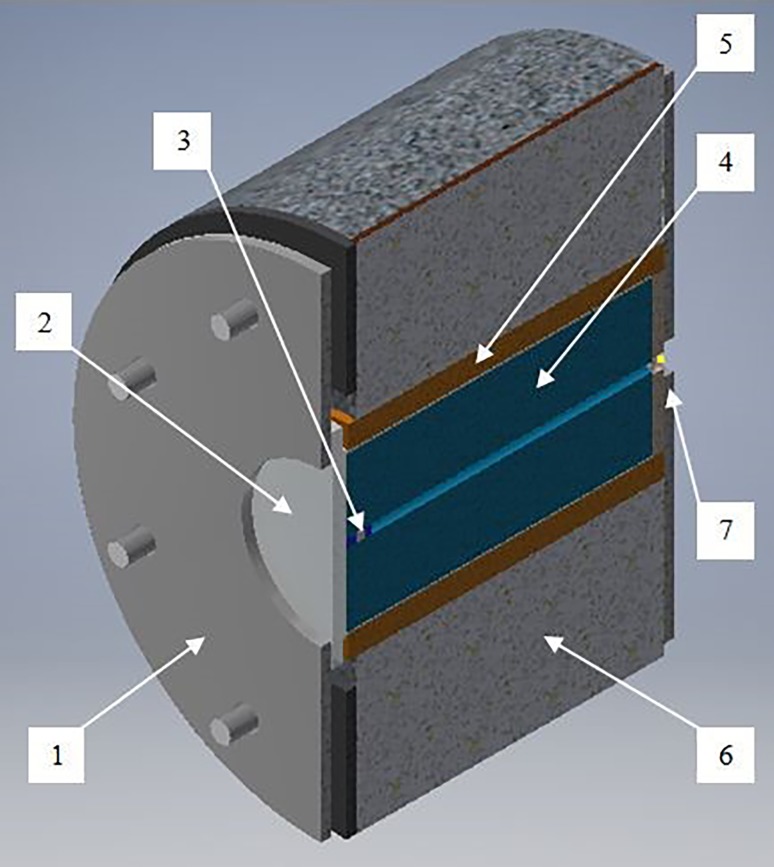
Prepared blast chamber (axial cross-section) (*1*—Frontal end metal plate; *2*—Protective window; *3*—Borehole with stemming; *4*—Blast cylinder; *5*—Damping layer; *6*—Chamber segment; *7*—Rear-end metal plate)

The blast chamber (Fig. 5) includes four concrete segments, axially connected with two metal plates. The segments are designed to radially move about 5 mm during the blast, acting as ‘impulse traps’ [17]. The rear metal plate includes an opening for inserting the cord. The frontal metal plate includes an opening, allowing filming of the frontal end face through a protective polycarbonate window.

The filming [13] captures crack development at the end face following the detonation. The cracking at the end face starts about when the detonation front reaches the stemming plug, which is seen as slight movement of the plug and occasionally a dimmed detonation-flash around it.

**Fig. 6:**
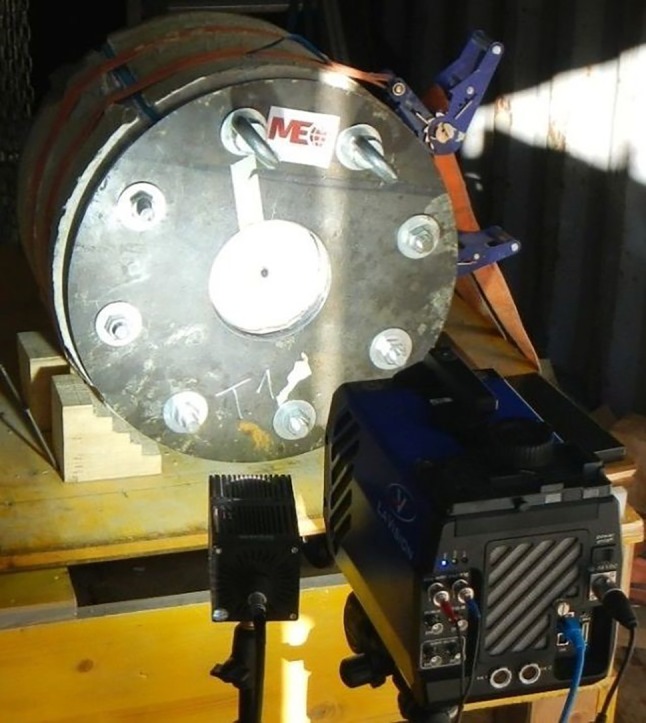
High-speed filming set-up for the blast tests

The filming set-up (Fig. 6) records the dynamic crack development, in most cases, with 24,656 fps at 336 × 336 pixels.

### Numerical Modelling

#### Modelling in Abaqus

Numerical modelling of blast fragmentation was done using the finite element method (FEM) and the discrete element method (DEM) [14]. The FEM approach (Abaqus) is suitable for modelling blast-induced damage, though presently quite limited for fragmentation analysis [14].

#### HiDEM Model

Blast cylinders are modelled with a 3D discrete element code (HiDEM) [11, 18]. A dynamic sedimentation method is used to generate the initial random structure of the model composed of rigid spheres of 2‑mm and 3‑mm diameter. Contacts between the particles are modelled using massless beams. The interaction potential between two particles is defined by the Euler-Bernoulli (EB) beam model. Estimates of the beam elastic energy are provided in [18]. The beams break due to excerted tension, shear, or bending beyond the fracture limit (Fig. 4 in [18]).

The particle-motion equation is given in [14]. The model assumes elastic-material behaviour. The stiffness matrix for linear-elastic EB beams under small deformation is provided in [18]. The modelled material has an elastic modulus of E = 19.7 GPa and a Poisson’s ratio of ν = 0.19, which is somewhat representative of the blast-test mortar. The stochastic mechanical behaviour of granular disordered materials was modelled using beams with reduced stiffness. These beams were randomly selected to have their stiffness reduced to 10% of the original value. Here, the fracture criterion [11, 18] was described by the elastic-strain threshold ε_crit_ = 0.0003.

The modelled mortar cylinders are Ø140 × 280 mm in size with a Ø10-mm borehole. The blast loading is radially applied onto the borehole wall according to a pressure-time function [14]. The modelled VOD is the same as in the blast tests. A simplified post-peak pressure drives all particles outwards with P_post_ = 0.0025P_peak_. The modelling uses three peak pressures of 166 MPa, 85 MPa, and 35 MPa, equivalent to 20 g/m, 12 g/m, and 6 g/m of PETN [14]. In addition, 20 mm of stemming was included. Quiet boundary conditions are applied to the mantle to avoid cracking due to reflected tensile waves.

#### Simplified n(s) Model

When blasted, the cylinder expands radially, inducing tangential tension and tensile cracks. In the crushing process, fragments are broken by continual shear deformation [19]. Such a process has a power-law FSD n_crush_(s)ds = C_1_s^-β^ds [11], where C_1_ is a constant and β indicates the degree of crushing/grinding, being β ~ 1.8–3.5 when dimension D = 3 [11, 15]. Dimensionless size s is measured in number of grains composing a fragment [15].

The dynamic tensile cracks can easily become unstable, branch, and further merge, forming fragments. This inherently-universal process leads to a characteristic FSD [11, 20]. The number of fragments n_bm_(s) of size s in an interval ds can be written as n_bm_(s)ds = C_2_s^-α^ exp(-s/C_3_)ds with α = (2D−1)/D, where C_2_ and C_3_ are non-universal constants [11, 15].

If n(s) describes the number-density of fragments with s number of grains, the FSD, or the number of fragments in a size-interval ds, can then be written as [15]:

1 $$\mathrm{n}(\mathrm{s})\mathrm{ds}\,=\mathrm{C}_{1}\mathrm{s}^{-\upbeta }\mathrm{ds}\,+\mathrm{C}_{2}\mathrm{s}^{\ -\upalpha }e\mathrm{xp}(-\mathrm{s}/\mathrm{C}_{3})\mathrm{ds}+\mathrm{n}_{\mathrm{b}}(\mathrm{s})\mathrm{ds}$$

where the boulder intensity n_b_ is given by the characteristic boulder size s_b_ and

2 $$\mathrm{n}_{\mathrm{b}}(\mathrm{s})\,=\,\exp (-\mathrm{s}/\mathrm{s}_{\mathrm{b}})$$

With the proper transformation from s to r, i. e. ds ∝ r^2^ dr for D = 3, leaving the exponential part of the second term in the n(s) formula and integrating the n(s), the mass passing fraction at screen size r (MPF(r)) can be approximated for fragments smaller than boulders [15]:

3 $$\mathrm{MPF}(\mathrm{r})=\mathrm{f}_{\mathrm{cr}}[1.0-\left(\mathrm{r}/\mathrm{r}_{\text{grain}}\right)^{(-3\upbeta +6)}]+\mathrm{f}_{\mathrm{bm}}(\mathrm{r}/{\mathrm{r}_{\max }})^{(-2\upalpha +6)}$$

where f_bm_ and f_cr_ determine the mass fraction of fragments formed in the branching-merging and the crushing process, r_grain_ is the approximated diameter of the material grain size, and r_max_ is the approximated diameter of the largest fragment.

## Preliminary Results

### Crack Patterns

**Fig. 7:**
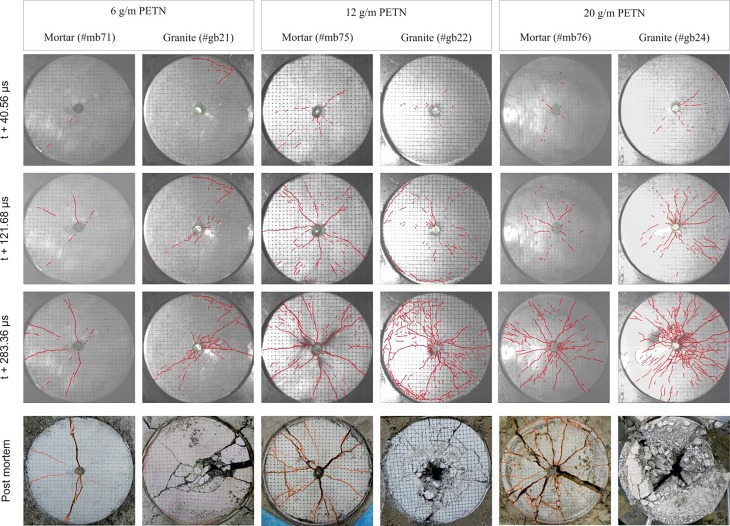
High-speed and post-mortem end-face images of the cylinders with respect to the charge and material

The high-speed images (Fig. 7) show more intensive cracking with the increase of charge. Similarly, crack patterns are denser and develop earlier in granite than in mortar. The high-speed images of both mortar and granite shots show three phases of crack development [13].

**Fig. 8:**
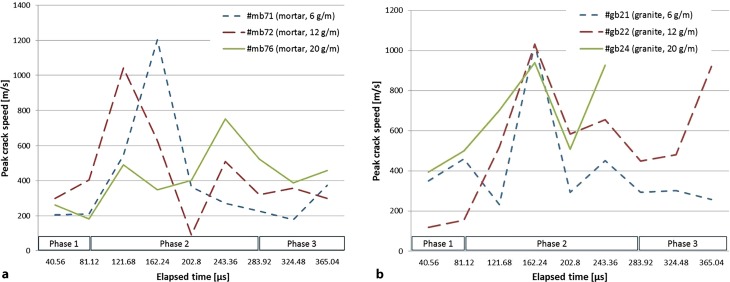
Peak crack speed in blast cylinders with respect to material, charge amount, and elapsed time (**a** Mortar curve set; **b** Granite curve set)

Firstly, following the plug movement, initial cracks emerge and propagate mainly in the radial direction. In the second phase, the crack-propagation speed reaches its peak and quickly drops with multiple cracks branching-merging (Fig. 8). After the second phase, the main cracks have reached the borehole and/or the mantle and end-face spalling with fumes leakage may occur.

The post-mortem crack patterns are observed at the end face (Fig. 7), on the mantle, and internally through computer tomography (CT) [13].

**Fig. 9:**
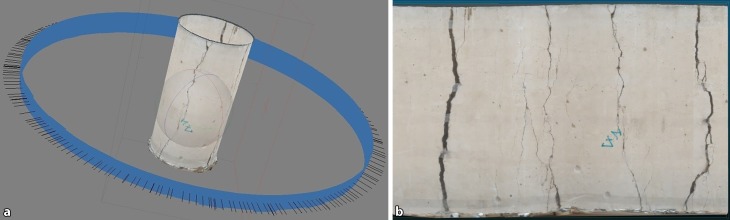
Post-mortem mantle image (**a** Processing; **b** “Unfolded” mantle, cylinder #mb71)

The mantle crack patterns are firstly photographed and then processed in Agisoft PhotoScan® to produce a 3D model of the mantle, which is then projected onto a 2D image for further analysis (Fig. 9).

So far the CT cross-sections and mantle images indicated that the number of main cracks and the number of main intersections basically do not change in the axial direction [13].

**Fig. 10:**
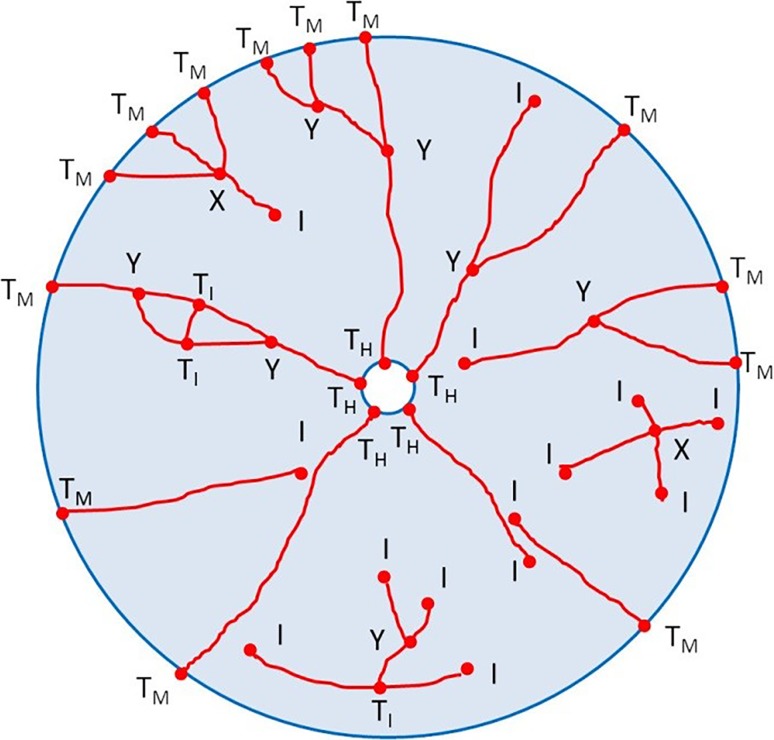
Topological representation of crack-pattern features

The crack patterns in the high-speed and post-mortem images are traced and topologically analysed, similarly as in [21]. This includes decomposition of a crack network into topological features (Fig. 10): branches, crack intersections (J_int_: X, Y, and T_I_), borehole intersections (T_H_), mantle intersections (T_M_), and crack-end nodes (I).

**Fig. 11:**
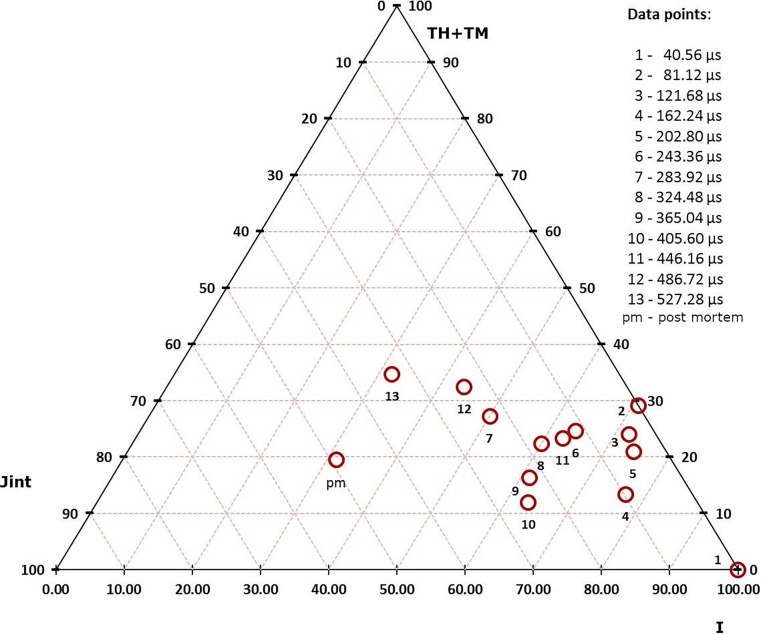
Results from the topological analysis (cylinder #mb75); the data points relate to crack-pattern state with respect to elapsed time

The analysis quantifies the development of the features in the images with respect to time, from 40.56 μs to 527.28 μs and finally to the post-mortem state (pm). The results are presented in a ternary diagram with respect to the percentage of the feature categories (Fig. 11).

The results show that the percentage of crack intersections rapidly increases and the percentage of end nodes drops during the second phase, as the initial smaller cracks coalesce.

The number of active end nodes (i. e. propagating crack tips) and intersections both increase with the increase of charge and more so in granite than in mortar.

**Fig. 12:**
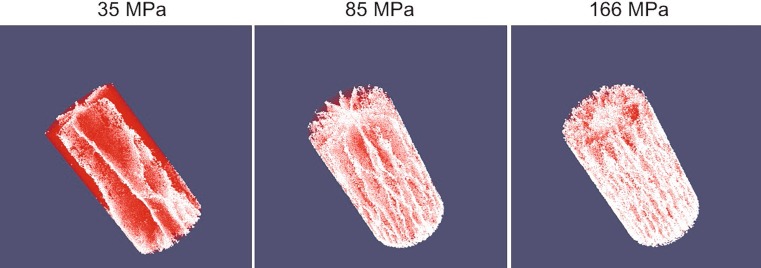
Crack patterns of modelled cylinders

Fig. 12 shows resulting 3D crack networks from the modelling with respect to the three loading levels at t = 400 µs. By increasing the loading level, the internal damage intensity increases. Accordingly, the crack system becomes more complex, increasing in number of main radial cracks and intersections.

### Fragmentation Analysis of Blasted Cylinders

**Fig. 13:**
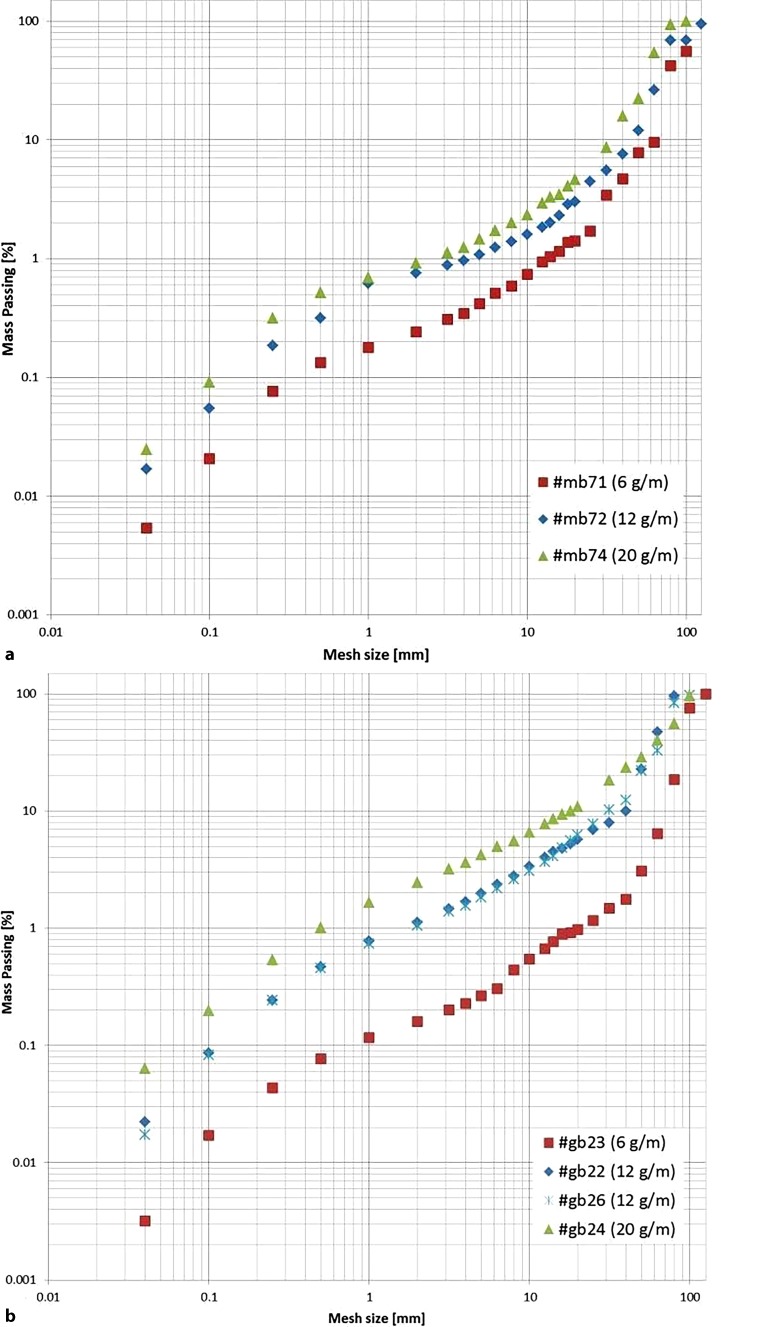
Selected sieving curves of blasted cylinders (**a** Mortar curve set; **b** Granite curve set)

Fig. 13 shows sieving FSD curves of selected blast cylinders. The curves shift upwards to contain larger fractions of fines when the charge is increased, in accordance with the Natural Breakage Characteristics (NBC) properties [4]. The curve-fitting procedure is ongoing.

### Numerical Estimates of Fragmentation and Fines Sources

**Fig. 14:**
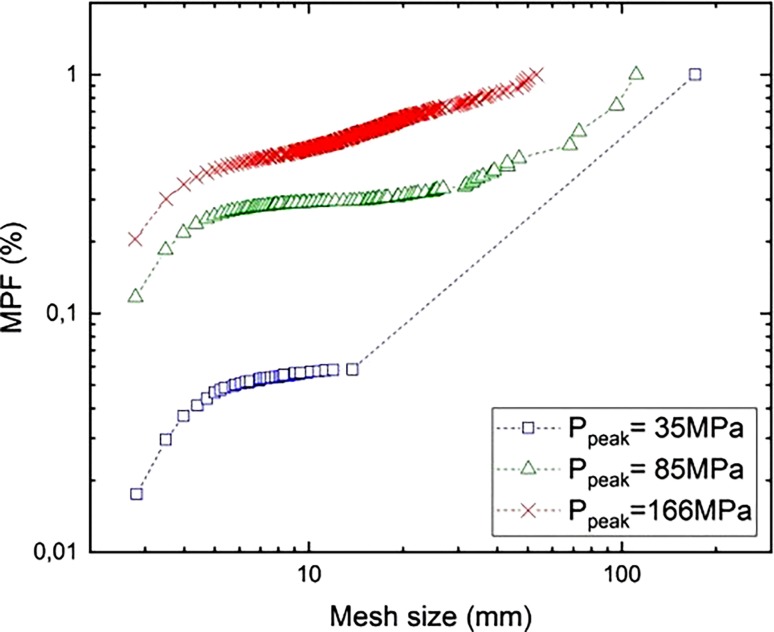
FSD curves of modelled cylinders

Fig. 14 shows FSD curves obtained with HiDEM modelling. A fragment is defined by the number of connected particles *N* and the screen size is that of the diameter of a volume-equivalent sphere. The curves represent the mass-passing fraction of the model for different blast-loading levels. The curve with P_peak_ = 166 MPa is similar to the sieving curves and well approximated by the Swebrec function [22]. The FSD curves from the DEM simulations also show an NBC-like relationship.

At 35 MPa and 85 MPa, the mass-passing curves indicate the “dust and boulders” phenomenon. The curve with P_peak_ = 35 MPa contains 94% of the cylinder mass in one boulder. The curve with P_peak_ = 85 MPa contains a wider range of fragment sizes and four boulders, with almost 50% of the cylinder mass. This results from blasting below the critical charge level [9, 12].

By using Eq. 3, the percentage mass fraction of fragments formed by branching-merging and crushing [15] can be determined for the results (Table 2). The size of the branching-merging fragments ranges from a single DEM particle to a maximum value, which depends on the applied loading level. Similar to the MPF, the size of these fragments is approximated by the diameter of the volume equivalent sphere.

**TABLE 2:** Percentage mass fraction of fragments formed by crushing, branching-merging, and in boulders

P_peak_ [MPa]	Fragment mass percentage [%]				Max. diameter of branching-merging fragments r_max_ [mm]
	Crushing	Branching-merging	Boulders	Branching-merging (<10 particles)	
35	4.5	1.5	94	0.5	14.1
85	26.5	19.6	53.9	1.1	70.9
166	37.5	55.3	7.1	4.3	52.4

**Fig. 15:**
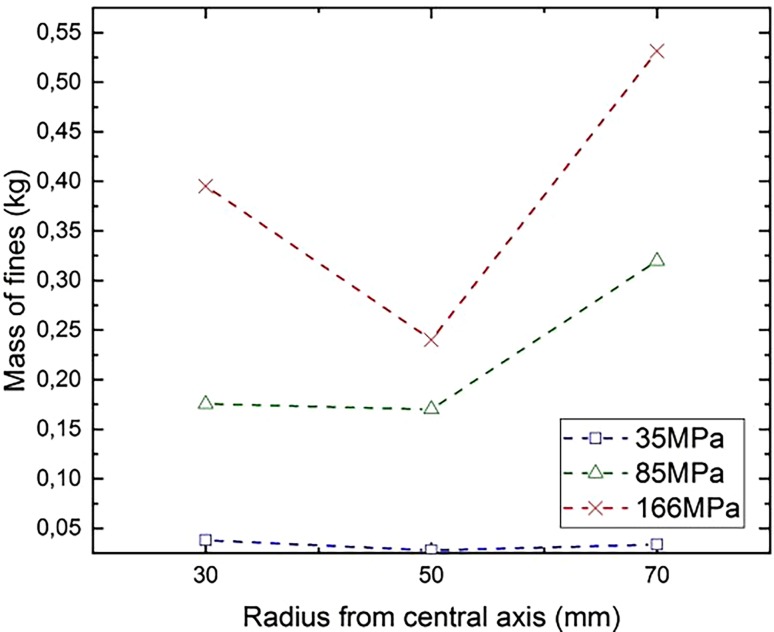
Absolute mass of fine-particles with respect to the radius from the borehole

Simulations have also been conducted with radially-layered mortar cylinders, like those in [8]. The cylinders are banded at radii r = 30 mm and r = 50 mm, creating three concentric regions. A routine calculates the absolute mass of very fine particles containing only one DEM particle in each region. Fig. 15 shows that the absolute mass of the fines in region 3 (50 mm < r < 70 mm) is larger than in region 1 (5 mm < r < 30 mm), thus confirming the results from [8].

## Conclusions

The ongoing project studies dynamic mechanisms behind blast-induced fines.

The filming shows a three-phase crack-pattern development. The main cracks and intersections in mortar are more numerous and appear earlier with higher charge and the same tendencies are observed in granite, but at a higher level. They are detectable before the third phase and do not significantly change in the axial direction. Although the crack speed is higher in mortar [13], crack patterns develop with more propagating crack tips in granite.

The numerical modelling has used the finite element method (Abaqus) and the discrete element method (HiDEM) with explicit time integration to model the dynamic crack propagation, branching and merging, and blast fragmentation of mortar cylinders. The FEM simulations provided results on dynamic 2D crack propagation, whereas the 3D behaviour has been more successfully simulated with the DEM code (HiDEM).

The HiDEM code provides realistic FSD results of blasted mortar cylinders, focusing on three major fragmentation mechanisms: borehole crushing, branching-merging, and secondary crushing of branching-merging fragments. The modelling results are in general agreement with the layered-cylinder blast results [8].

The FSD sieving curves of both blasted and modelled cylinders follow reasonably well the NBC parallel upward shift with the charge increase.

Future work will include further topological analysis of the images, analysis of blast-induced 3D crack patterns, determining other possible fines-generating mechanisms acting in the high-speed images, and further fragmentation analysis of the modelled and the blasted cylinders, including laser diffractometry for the grain sizes below 40 μm. The comparison of the simulation results with the blast-test results will take place after necessary calibrations. The final results will determine the relative influence of observed dynamic mechanisms on the fines generation and a discussion of how the fines could be suppressed.
